# The Connection of Monocytes and Reactive Oxygen Species in Pain

**DOI:** 10.1371/journal.pone.0063564

**Published:** 2013-05-02

**Authors:** Dagmar Hackel, Diana Pflücke, Annick Neumann, Johannes Viebahn, Shaaban Mousa, Erhard Wischmeyer, Norbert Roewer, Alexander Brack, Heike Lydia Rittner

**Affiliations:** 1 Klinik und Poliklinik für Anaesthesiologie, Universitätsklinikum, Würzburg, Germany; 2 Klinik für Anesthaesiologie m. S. operative Intensivmedizin, Campus Benjamin Franklin, Charité – Universitätsmedizin Berlin, Germany; 3 Institut für Physiologie, Universität Würzburg, Germany; 4 Klinik für Anaesthesiology, Campus Virchow, Charité – Universitätsmedizin Berlin, Germany; Southern Illinois University School of Medicine, United States of America

## Abstract

The interplay of specific leukocyte subpopulations, resident cells and proalgesic mediators results in pain in inflammation. Proalgesic mediators like reactive oxygen species (ROS) and downstream products elicit pain by stimulation of transient receptor potential (TRP) channels. The contribution of leukocyte subpopulations however is less clear. Local injection of neutrophilic chemokines elicits neutrophil recruitment but no hyperalgesia in rats. In meta-analyses the monocytic chemoattractant, CCL2 (monocyte chemoattractant protein-1; MCP-1), was identified as an important factor in the pathophysiology of human and animal pain. In this study, intraplantar injection of CCL2 elicited thermal and mechanical pain in Wistar but not in Dark Agouti (DA) rats, which lack p47^phox^, a part of the NADPH oxidase complex. Inflammatory hyperalgesia after complete Freund's adjuvant (CFA) as well as capsaicin-induced hyperalgesia and capsaicin-induced current flow in dorsal root ganglion neurons in DA were comparable to Wistar rats. Macrophages from DA expressed lower levels of CCR2 and thereby migrated less towards CCL2 and formed limited amounts of ROS *in vitro* and 4-hydroxynonenal (4-HNE) in the tissue in response to CCL2 compared to Wistar rats. Local adoptive transfer of peritoneal macrophages from Wistar but not from DA rats reconstituted CCL2-triggered hyperalgesia in leukocyte-depleted DA and Wistar rats. A pharmacological stimulator of ROS production (phytol) restored CCL2-induced hyperalgesia *in vivo* in DA rats. In Wistar rats, CCL2-induced hyperalgesia was completely blocked by superoxide dismutase (SOD), catalase or tempol. Likewise, inhibition of NADPH oxidase by apocynin reduced CCL2-elicited hyperalgesia but not CFA-induced inflammatory hyperalgesia. In summary, we provide a link between CCL2, CCR2 expression on macrophages, NADPH oxidase, ROS and the development CCL2-triggered hyperalgesia, which is different from CFA-induced hyperalgesia. The study further supports the impact of CCL2 and ROS as potential targets in pain therapy.

## Introduction

In inflammation leukocyte subpopulations may play different roles in the generation of hyperalgesia. Intraplantar injection of the neutrophilic chemokine CXCL2/3 (macrophage inflammatory protein, MIP-2) leads to a selective accumulation of neutrophils. However, in contrast to complete Freund's adjuvant induced (CFA) inflammation with similar numbers of neutrophils in the tissue, CXCL2/3 induces no mechanical or thermal hyperalgesia [1]. In the early phase of CFA inflammation, neutrophils tonically release opioid peptides resulting in basal analgesia, which could counterbalance proalgesic effects [2]. Therefore, other cell populations appear to be responsible for inflammatory hyperalgesia. Monocytes and macrophages are major contributors to inflammatory infiltrate in later phases of inflammation [3].

CCL2 is an important and well-characterized monocytic chemokine [4], [5], [6]. CCL2 is a critical player in neuropathic pain and might be important to inflammatory pain [6], [7]. Injection of CCL2 in the paw elicits thermal and mechanical hyperalgesia [8]. Furthermore, exposure of macrophages to CCL2 results in the release of reactive oxygen species (ROS), proinflammatory cytokines (e.g. IL-1, TNF-α, MCP-1) and profibrotic growth factors (e.g. PDGF, TGF-β) [9], [10]. The phagocyte NADPH oxidase complex generates ROS. ROS play a role in the pathogenesis of acute and chronic pain and have been postulated as mediators of inflammatory [11] and neuropathic pain e.g. chemotherapy-induced neuropathic pain [12]. ROS-induced oxidative stress during inflammation results in highly reactive lipid peroxidation products like 4-hydroxynonenal (4-HNE) protein adducts [13]. Potential targets of ROS are transient receptor potential vannilloid 1 (TRPV1) or transient receptor potential ankyrin 1 (TRPA1) expressed on nociceptors.

ROS production by the phagocyte NADPH oxidase complex is achieved by two catalytic domains including gp91^phox^ and p22^phox^ a regulatory domain containing p40^phox^, p67^phox^ and p47^phox^, coded by *ncf1*. NADPH oxidase can be inhibited by apocynin [14]. The NOX2 complex (the phagocyte NADPH oxidase) yields superoxide anion, which is further transformed into hydrogen peroxide and hydroxyl radicals [15]. Superoxide dismutase (SOD) catalyses the transformation of superoxide into oxygen and hydrogen peroxide. Catalase catalyses the decomposition of hydrogen peroxide to water and oxygen [16]. Thus, they are an important antioxidant defense in nearly all cells exposed to ROS. Dark Agouti (DA) rats develop an increased susceptibility to arthritis due to a genetic polymorphism for *ncf1* encoding p47^phox^ [17]. This variant of *ncf1* results in a reduced release of ROS from all leukocyte populations including peritoneal macrophages [18]. As a consequence macrophages are not able to suppress the T-cell response, which in turn leads to an increased arthritis [17]. In comparison, Wistar rats are less susceptible to adjuvant-induced arthritis [19].

The present study examines the question whether the formation of ROS from monocytes is important for the development of CCL2-induced hyperalgesia. We compared DA rats with reduced activity of NADPH oxidase to Wistar rats with normal NADPH oxidase. Specifically we investigated 1) the contribution of macrophages and ROS to CCL2-induced hyperalgesia using cross over adoptive transfer experiments in DA and Wistar rats 2) inflammatory pain and TRPV1 responsiveness in both strains 3) CCR2 expression, leukocyte migration and ROS and HNE generation in response to CCL2 in both strains and 4) specific role of ROS and NADPH oxidase in CCL2-induced hyperalgesia in Wistar rats.

## Materials and Methods

### Animals

Animal protocols were approved by the animal care committees (Landesamt für Arbeitsschutz, Gesundheitsschutz und Technische Sicherheit, Senate of Berlin and Regierung Unterfranken, both Germany), and are in accordance with the International Association for the Study of Pain [20]. Male Wistar and DA rats weighing 180–220 g were treated as described below under brief isoflurane anesthesia. Animals were sacrificed using intracardial injection of a solution of T61 (embutramide, mebezonium and tetracaine) under isoflurane anesthesia according to national guidelines.

### Measurement of nociceptive thresholds

Mechanical thresholds were determined using the paw pressure algesiometer (modified Randall-Selitto test; Ugo Basile, Comerio, Italy) as described before [1]. The pressure required to elicit paw withdrawal using a blunt piston onto the dorsal surface of the hind paw, the paw pressure threshold was determined. Treatments were randomized, and the experimenter was blinded to the treatments. A decrease in the paw pressure threshold was interpreted as pain (hyperalgesia) whereas a rise in the paw pressure threshold was interpreted as analgesia (antinociception).

Thermal nociceptive thresholds were measured by the Hargreaves test as previously described [1]. The latency (time; s) required to elicit paw withdrawal was measured with an electronic timer (IITC Inc/Life Science, Woodland Hills, CA; USA) after application of radiant heat to the plantar surface of a hind paw from underneath the glass floor with a high-intensity light bulb. The stimulus intensity was adjusted to 12 or 20 s for the paw withdrawal latency (PWL) in noninflamed paws, and a cutoff of 30 s was set to avoid tissue damage. Different baseline latencies as previously described in the literature [21] were used due to requirements of the local animal welfare authorities. The average of two measurements taken with 20 s intervals was calculated. A decrease in paw withdrawal latency was interpreted as pain (hyperalgesia) whereas a rise in paw withdrawal latency was interpreted as analgesia (antinociception). To better compare data with different baselines data are shown in %MPE (%MPE  =  [PWL_treated_−PWL_pretreated_]/[cutoff−PW _pretreated_])

DA and Wistar rats were i.pl. injected into the right hind paws with several doses of CCL2 (0.3–3 µg in 100 µl) (Peprotech, Hamburg, Germany) and mechanical as well as thermal pain thresholds were measured after different time points (1–24 h). Doses were chosen according to former studies and pilot experiments [22], [23]. Likewise, 150 µl complete Freund's adjuvant (CFA, Calbiochem, Darmstadt, Germany) was injected i.pl. to measure thermal and mechanical pain thresholds after 2–48 h. In DA rats, thermal pain thresholds were measured 15–120 min after i.pl. injection of capsaicin (Sigma Aldrich) (30 µg in 100 µl 1% EtOH) [24]. In the next set of experiments, Wistar rats were treated with different doses of antioxidants like SOD (3–300 U i.pl.), catalase (2–500 U i.pl.), tempol (1.5–15 mg i.p.) or apocynin (1 µg i.pl.) (all Sigma Aldrich, Heidelberg, Germany) coinjected or given 15 min before 3 µg CCL2 i.pl. Doses were chosen according to the literature [11], [14], [16]. Mechanical pain thresholds were measured after 0, 1 or 3 h.

### Immunodepletion and adoptive transfer

Recipient rats were treated with cyclophosphamide (Baxter, Unterschleissheim, Germany) according to a protocol established in our laboratory [25]. Briefly, the animals were injected intraperitonially (i.p.) three days and one day before the experiment with 100 mg/kg and 50 mg/kg of cyclophosphamide, respectively. This treatment results in over 90% reduction in circulating leukocytes. For adoptive transfer different numbers of macrophages from peritoneal macrophages from Wistar or DA rats (see below) were injected intraplantarly (i.pl.) 30 min prior to i.pl. injection of 3 µg CCL2 each in 50 µl solvent (0.9% NaCl) as described before for adoptive transfer of neutrophils [23].

### Phytol treatment of rats

To reconstitute ROS formation DA rats were injected subcutaneously (s.c.) with 200 µl 3,7,11,15-Tetramethyl-2-hexandecen-1-ol (phytol, Sigma Aldrich, Heidelberg, Germany) 5 d prior to i.pl. injection of CCL2 (0.3–3 µg in 100 µl) [26].

### Monocytes/macrophages isolation from rats

Peritoneal monocytes/macrophages were harvested 4 d after i.p. injection of 3 % thioglycollate in phosphate buffered saline [1]. Rats were sacrificed and the peritoneum lavaged with PBS/EDTA (all Sigma Aldrich). Monocytes/macrophages were >95 % pure as confirmed by flow cytometry.

### Real-time reverse transcription-polymerase chain reaction (RT-PCR) for CCR2 mRNA

Total RNA from peritoneal macrophages from DA and Wistar rats was extracted using a commercially available kit (NucleoSpin RNA II, Macherey-Nagel, Dueren, Germany). RNA from macrophages was reversely transcribed into cDNA using the High-Capacity cDNA Reverse Transcription Kit (Applied Biosystems/Life Technologies, Carlsbad, CA, USA). cDNA from peritoneal macrophages was amplified with TaqMan Gene Expression Assay for CCR2 and GAPDH (Rn00573193_s1, Rn 0177576_g1, Applied Biosystems). The cycling conditions were: 50 cycles, annealing and elongation 1 min at 60°C. Mean Ct value of all samples was 26.8 for CCR2 mRNA and 22.8 for GAPDH mRNA. Results were calculated using the 2-ddCT method for relative quantification.

### Chemotaxis assay

CCL2 dissolved in sterile Hanks balanced salt solution/0.5% bovine serum albumin (Sigma Aldrich) was placed in the lower well of a Boyden chamber (Costar, Amsterdam, Netherlands) and 2×10^6^ rat peritoneal macrophages from DA and Wistar rats in the upper well. After 2 h incubation at 37°C and 5% CO_2_ the number of cells in the cell suspension from the lower well was quantified by flow cytometry using fluorescent beads (see below). The migration index was calculated by dividing the number of cells migrating towards CCL2 by the number of migrating cells using Hanks balanced salt solution/bovine serum albumin alone [27], [28].

### Flow cytometry – leukocyte subpopulations

Cellular staining in the subcutaneous paw tissue was performed as previously described [1]. The right hind paws of either Wistar rats or DA rats were injected with 100 µl 0.9 % NaCl or 3 µg CCL2 (each for 3 h) or with 150 µl CFA (for 2 h) as positive control. Animals were sacrificed and paw tissues were prepared for FACS analysis.

Briefly, after enzymatic digestion and permeabilization, single cell suspensions were incubated with the following primary and secondary antibodies (all antibodies by BD Biosciences, Heidelberg, Germany unless stated otherwise): mouse anti rat CD45 phycoerythrin (PE) Cy5 monoclonal antibody identifies all hematopoetic cells, 4 µg/ml), mouse anti-rat ED1 fluorescein (FITC, recognizing the CD68 antigen expressed on monocytes/macrophages, 2 µg/ml, Serotec, Düsseldorf, Germany). Absolute numbers of cells per paw in the stained cell suspensions were calculated using fluorescent TruCOUNT® beads. The FACS Scan acquired 20.000–70.000 FACS events. Data analysis used CellQuest software (all BD Biosciences).

Cell quantification after cyclophosphamide (CTX) immune depletion was analyzed in peripheral blood from donor rats collected through heart puncture. 100 µl of blood were lysed using 2 ml FACS Lysing Solution (BD Bioscience) and suspended in PBS. One the verge of measurements cell suspensions were mixed with TruCOUNT® beads (BD Biosciences) and analyzed as described before [27].

### Formation of ROS in peritoneal macrophages

1×10^6^ macrophages were first stimulated with CCL2 in different concentrations (2 µg/ml and 0.5 µg/ml) and then incubated with dihydrorhodamine 123 (DHR 123, Phagoburst® (Bursttest), Orpegen Pharma, Heidelberg, Germany) which becomes fluorescent when oxidized. ROS formation was measured after 1 h. Phorbol 12-myristate 13-acetate (PMA) was used as a positive control. Fluorescence was determined by flow cytometry and the geometric mean was obtained.

### Immunofluorescence

Rats were deeply anesthetized with isoflurane and perfused transcardially with 0.1 mM PBS, pH 7.4, and with cold PBS containing 4% paraformaldehyde pH 7,4 (fixative solution). The subcutaneous tissue was dissected from hind paws, postfixed in the fixative solution, cryoprotected in 15% sucrose solution at 4°C overnight, embedded in tissue-Tek compound (OCT, Miles Inc., Elkhart, IN), and frozen. Seven-micrometer-thick sections were prepared on cryostat and mounted on gelatin-coated slides.

For immunostaining, the tissue sections were incubated with a) polyclonal rabbit anti-HNE (Biotrend, Cologne, Germany) in combination with mouse monoclonal anti-ED1 (CD68, Serotec, Oxford, Great Britain) overnight. The tissue sections were washed with PBS and then incubated with the appropriate secondary antibodies: Texas red conjugated goat anti-rabbit antibody in combination with FITC conjugated donkey anti-mouse antibody. Finally, the tissues were washed in PBS, stained with 4′,6 diamidino-2-phenylindole (DAPI) and mounted on vectashield (Vector Laboratories, Burlingame, CA). To demonstrate specificity of staining, the following controls were included: omission of either the primary antibodies or the secondary antibodies [29].

### Enzyme-linked immunosorbent assay (ELISA)

4-HNE or HNE is a well-known by-product of lipid peroxidation after the generation of ROS and is widely accepted as a stable marker for oxidative stress. For measurement of HNE subcutaneous paw tissue was obtained and minced in 20 mM Tris buffer. After centrifugation supernatant was used for quantification of total protein content in the paw tissue. Extracted protein was diluted in lysis buffer and incubated with bicinchoninic acid (BCA) protein assay reagent (Pierce) for quantification on a plate reader (Tecan). HNE content of paw tissue was measured by a commercially available kit (OxiSelect^TM^ HNE-His Adduct ELISA Kit, Cell Biolabs) according to the manufacturer's instruction. It measures HNE adducts to proteins.

### Electrophysiology

DRGs of the spinal segments L1-S1 from male adult Wistar and DA rats (200–250 g) were dissected, digested with collagenase and trypsin followed by a percoll purification. DRG neurons were plated on poly-L-lysine coated coverslips and cultured at 37°C and 5% CO2.in D-MEM supplemented with 10% horse serum, 1% penicillin-streptomycin and 0.5% L-glutamine. DRG neurons were incubated for 4 h prior to recording. Whole-cell recordings from DRG neurons were performed at room temperature [30]. Borosilicate glass pipettes (Science Products, Hofheim, Germany) had a resistance of 3–6 MΩ. Data were generated with an EPC 9-amplifier and HEKA-software (both HEKA, Lambrecht, Germany) and saved on an Apple G4 (Apple, Cupertino, CA, USA). Extracellular solution: NaCl 125 mM, KCl 5 mM, MgCl2 1 mM, CaCl_2_ 2 mM, D(+)-Glucose 10 mM, Hepes 25 mM, pH: 7.4. Pipette solution: NaCl 5 mM, KCl 140 mM, MgCl_2_ 2 mM, CaCl_2_ 0.0785 mM (free Ca^2+^ 0.001 mM), Hepes 10 mM, ATP 2 mM, EGTA 0.08 mM (all Merck or Sigma Chemicals), pH 7.24. Capsaicin (Sigma Chemicals) was diluted in extracellular solution and applied in ascending concentrations (0.5–1 µM). Membrane potential was clamped to −70 mV during current recordings. Whole cell recordings were done after application of capsaicin (0.5–1 µM).

### Statistical analysis

Data are presented as raw values or % maximal possible effect (%MPE) (mean ± SEM). Data were tested for normality and for equal variance. Normally distributed data were analyzed by t-test. If aliquots of one sample were exposed to different conditions or repeated measurements were taken, a Two Way repeated measurement (RM) ANOVA was used. The post hoc comparisons were performed by Student-Newman-Keuls. In case of not normally distributed data the test was performed on ranks. Differences were considered significant if p<0.05.

## Results

### CCL2-induced hyperalgesia in Wistar but not DA rats

**Figure 1:**
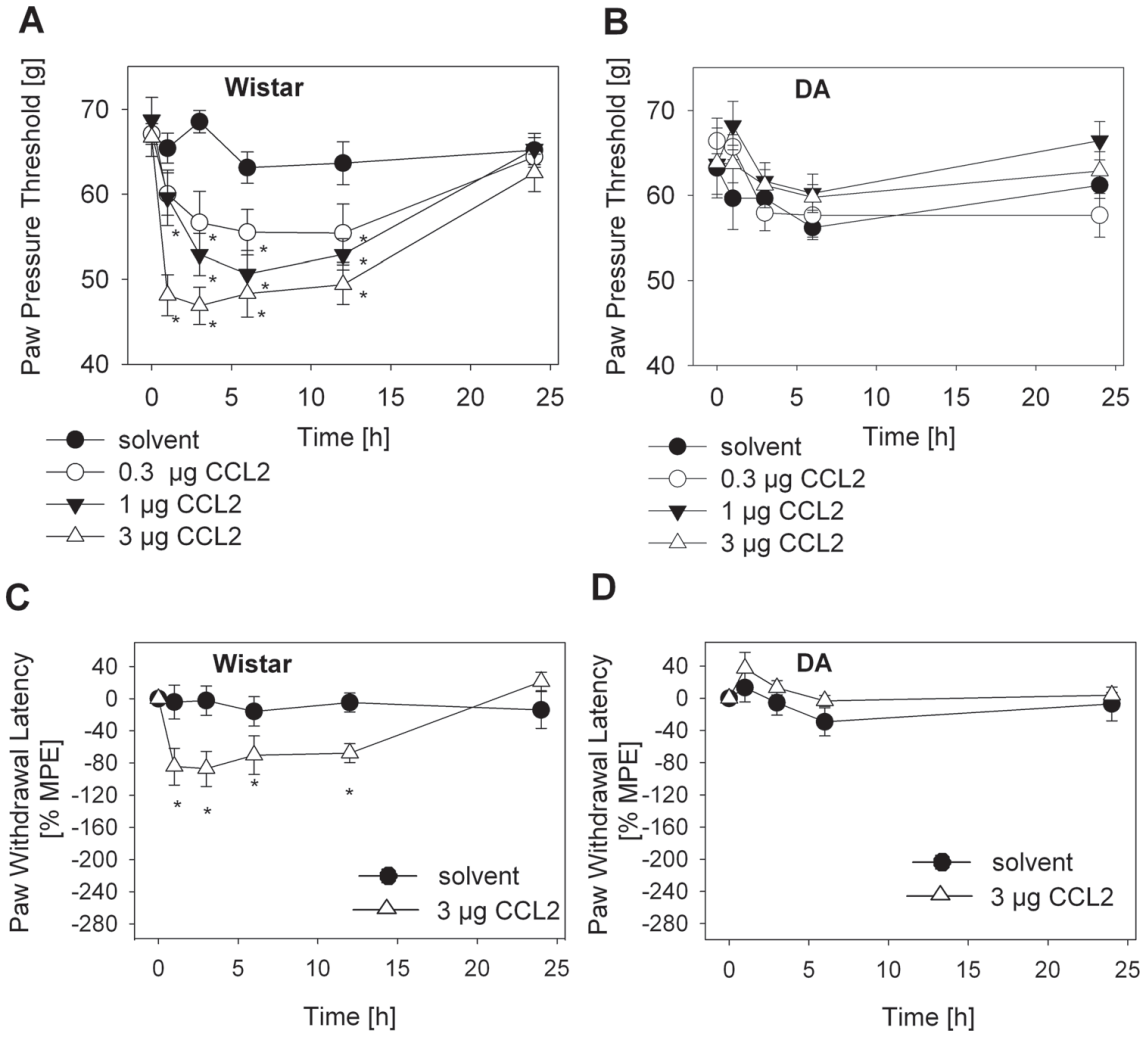
CCL2-induced hyperalgesia in Wistar, but not in Dark Agouti (DA) rats. Mechanical (**A, B**) and thermal (**C, D**) nociceptive thresholds were quantified before and after i.pl. injection of CCL2 (0.3 µg – open circle, 1 µg – filled triangle, 3 µg – open triangle) or 0.9 % NaCl (solvent  =  control – filled circle) into Wistar (**A, C**) and DA (**B, D**) rats (*p<0.05 versus time point 0 h, Two Way RM ANOVA, Student-Newman-Keuls, n = 6). Data are presented as means ± SEM of raw values (**A, B**) or % maximal possible effects (MPE) (**C, D**).

Injection of CCL2 into the hind paw of Wistar rats elicited a dose-dependent mechanical (**Fig. 1A**) and thermal (**Fig. 1C**) hyperalgesia between 1 and 12 h returning to baseline at 24 h. The maximal effect was seen at 3 h using 3 µg CCL2. This dose and time point was used for further experiments. In contrast, mechanical (**Fig. 1B**) and thermal (**Fig. 1D**) nociceptive thresholds were unchanged following CCL2 injection into NAPDH oxidase-deficient DA rats.

### Similar development of inflammatory hyperalgesia and TRPV1 responsiveness in DA compared to Wistar rats

**Figure 2:**
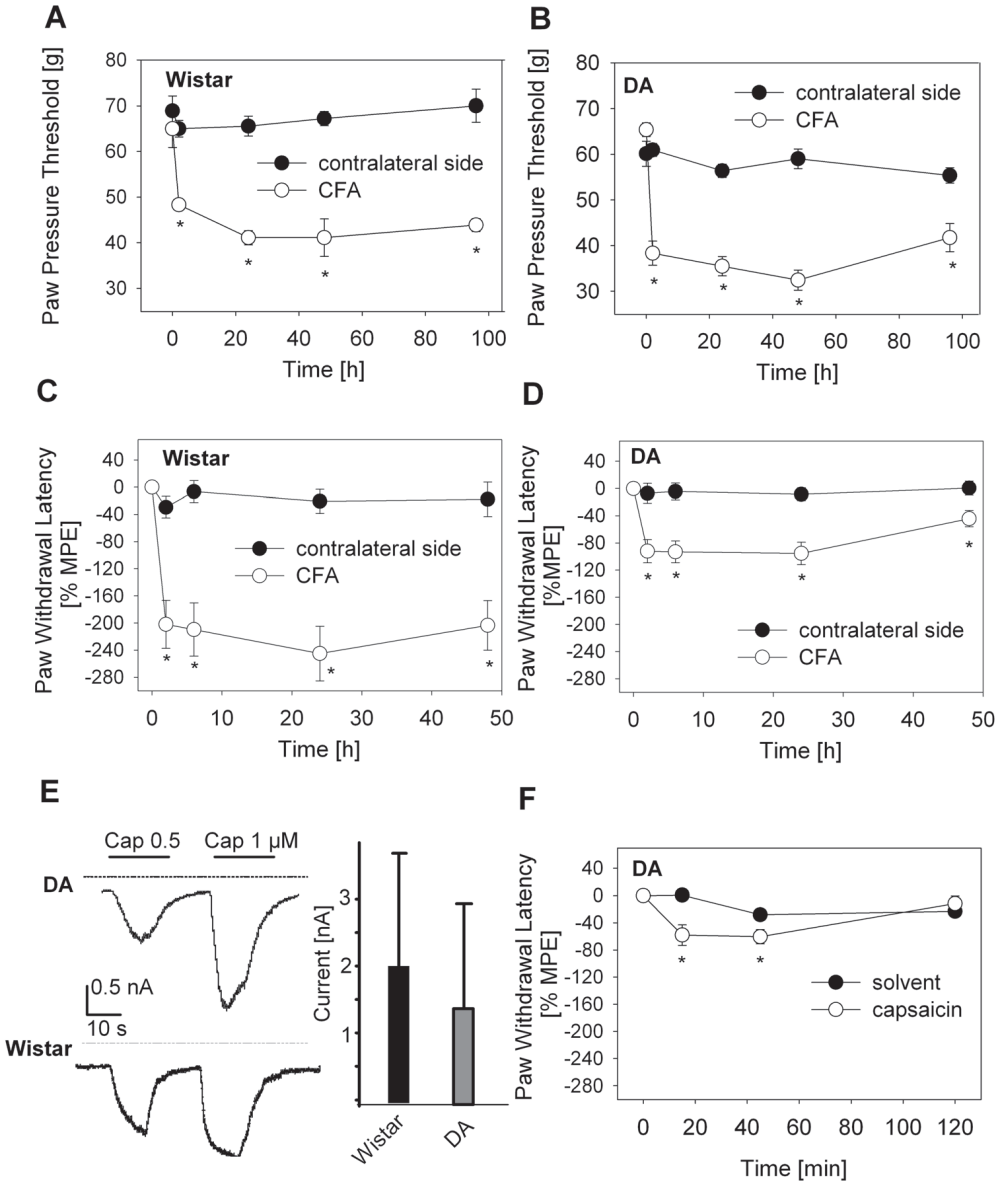
Comparable nociceptive thresholds and DRG currents in Wistar and Dark Agouti (DA) rats. Rats received an i.pl. injection of CFA (**A–D**; ipsilateral injected paw  =  open circle, contralateral, non injected paw  =  filled circle). Mechanical (**A, B**) and thermal nociceptive (**C, D,**) thresholds were quantified (*p<0.05 versus time point 0 h, Two Way RM ANOVA, Student-Newman-Keuls, n = 6). Data are presented as means ± SEM of raw values (**A, B**) or % maximal possible effects (MPE) (**C, D**). (**E**) DRG-neurons from naïve DA rats were obtained and cultured for 48 h. Representative traces of whole cell recordings are shown after application of capsaicin (500, 1000 nM, left). Bar graph quantifies capsaicin-activated inward currents in DRG neurons from Wistar and DA rats (right) (n = 9, means ± SEM, p>0.05, t- test). In addition DA rats received i.pl. injection of capsaicin (**F**; solvent – filled circle, 30 µg capsaicin – open circle) and thermal nociceptive thresholds were quantified (*p<0.05 versus time point 0 h, Two Way RM ANOVA, Student-Newman-Keuls, n = 6). Data are presented as means ± SEM of % maximal possible effects (MPE).

To test whether DA rats have a more generalized defect in nociception in response to inflammatory or proalgesic mediators, we determined nociceptive thresholds after an inflammatory stimulus (CFA). In DA rats mechanical and thermal nociceptive thresholds were lowered in after i.pl. CFA. The mechanical thresholds in DA rats were similar after CFA treatment compared to Wistar rats (**Fig. 2A, B**), while the decrease in thermal nociceptive thresholds after i.pl. injection of CFA was less pronounced in DA rats compared to Wistar rats (**Fig. 2C, D**). Thermal hyperalgesia after CFA (**Fig. 2C, D**) was more intense than after CCL2 (**Fig. 1C, D**) in both strains. *In vitro* treatment of cultured DRG neurons from DA rats and Wistar rats with capsaicin elicited comparable inward currents in whole cell recordings. There was a small but not significant difference in current amplitude (**Fig. 2E**). To test whether DA rats have a defect in TRPV1 function *in vivo* DA rats were treated i.pl. with capsaicin (**Fig. 2F**). Thermal thresholds after i.pl. capsaicin treatment were similar compared to Wistar rats as shown before [24]. In DA rats the threshold decreased from 10.0 +/− 0.6 s (BL) to 4.7 +/− 0.8 s (15 min) and in Wistar rats from 10.4 +/− 0.3 s (BL) to 4.9 +/− 0.2 s (15 min).

### Reduced CCR2 expression and macrophage chemotaxis in DA rats

**Figure 3:**
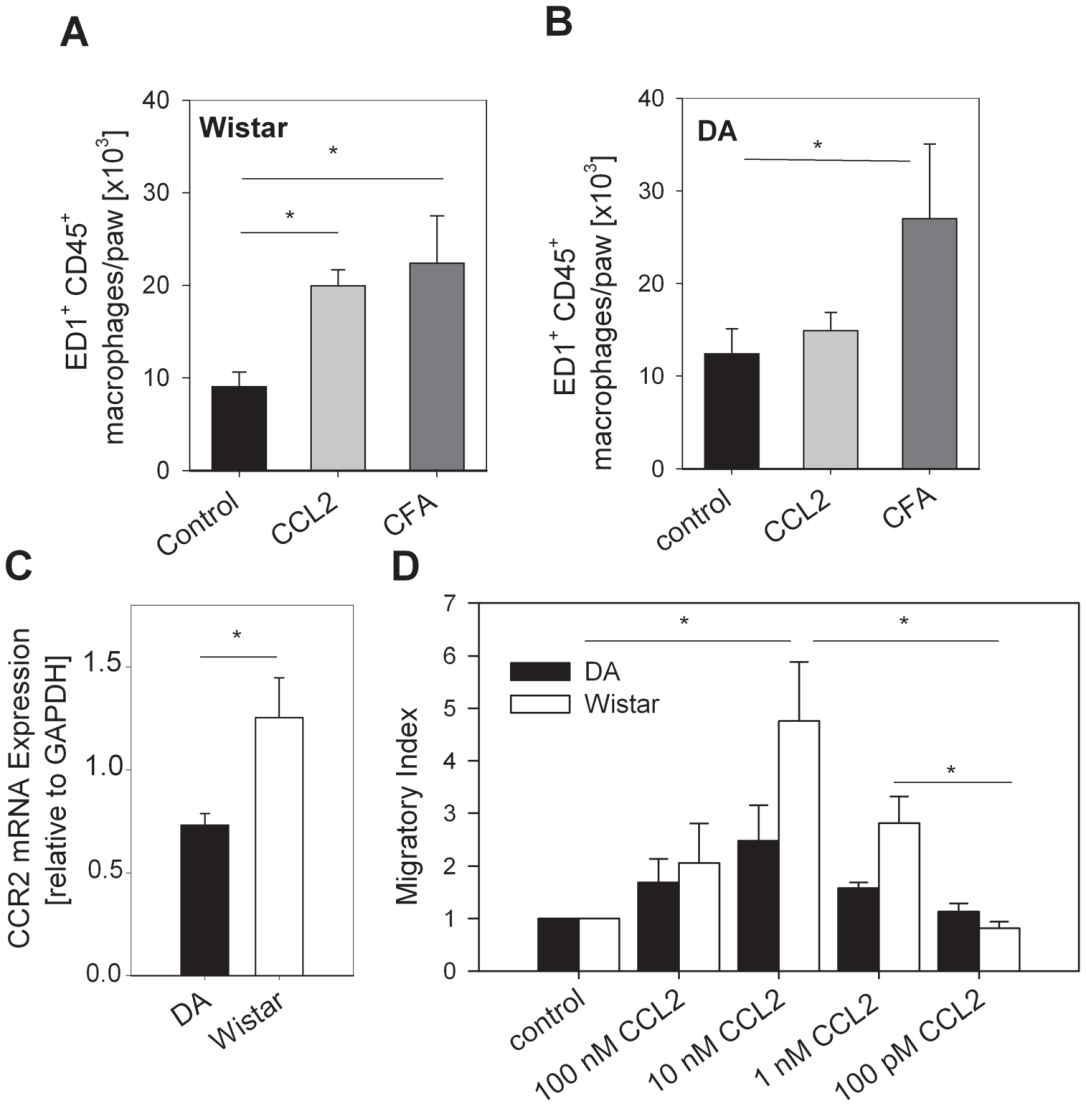
Defective CCL2-induced chemotaxis of macrophages from Dark Agouti (DA) in comparison to Wistar rats. Wistar (**A**) and DA (**B**) rats were i.pl. injected with 3 µg CCL2 (light grey bars), with CFA (dark grey bars, positive control) or with 0.9% NaCl (black bars, solvent negative control). Single cell suspensions were prepared from paw tissue and the number of infiltrating ED1^+^ CD45^+^ macrophages was quantified by flow cytometry (n = 4, *p<0.05, One Way ANOVA, Student-Newman-Keuls). (**C**) CCR2 mRNA from peritoneal rat macrophages from Wistar (white bars) as well as DA (black bars) rats was measured with real time PCR (*p<0.05, t-test, n = 6). (**D**) The migratory capacity of macrophages from Wistar rats (white bars, n = 8) and DA rats (black bars, n = 5) was quantified in a Boyden chamber in response to CCL2 and solvent control (p<0.05, One way ANOVA, Dunns-Method).

Local injection of chemokines results in recruitment of leukocytes. In order to explore the effectiveness of recruitment, the number of leukocytes in the inflammatory infiltrate was analyzed by flow cytometry. Intraplantar injection of CFA lead to the recruitment of similar numbers of ED1^+^ CD45^+^ macrophages into the paw of both DA and Wistar rats but CCL2 injection did not induce any macrophage recruitment in DA rats (**Fig. 3A, B**). To explore causes for the defect in recruitment, CCR2 expression and function was measured in peritoneal thioglycollate-elicited inflammatory macrophages. Macrophages from DA rats expressed small but significantly lower levels of CCR2 mRNA compared to Wistar rats (**Fig. 3C**). Thiogycollate-elicited peritoneal macrophage from Wistar rats migrated towards CCL2 *in vitro* with a maximal effect at 10 nM CCL2. In contrast, macrophage from DA rats migrated indeed but to a significantly lesser extent in response to CCL2 *in vitro* (**Fig. 3D**).

### Restoration of defective ROS production CCL2-induced hyperalgesia in DA rats

**Figure 4:**
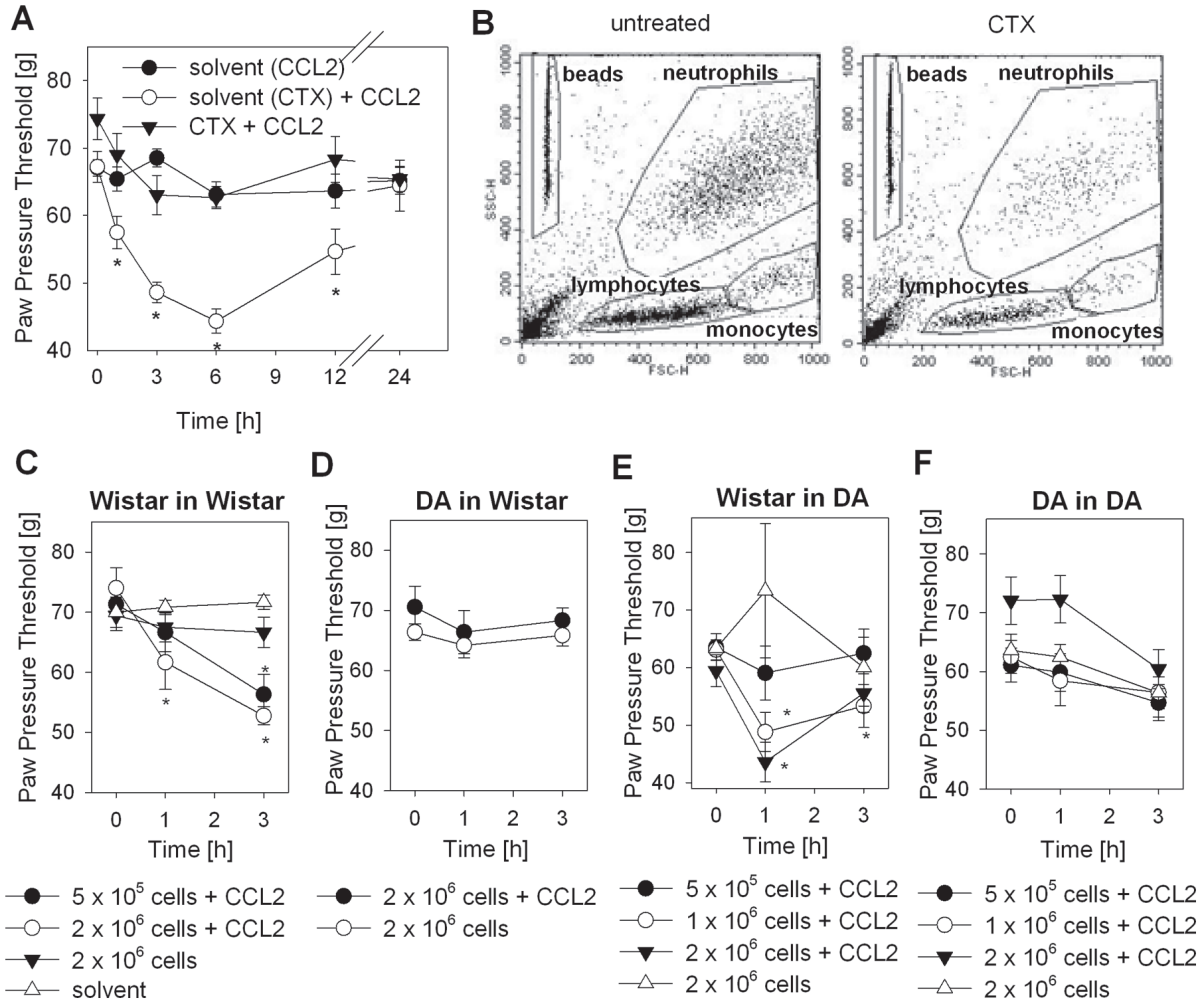
Reconstitution of CCL2-induced hyperalgesia by adoptive transfer of macrophages derived from Wistar, but not from Dark Agouti (DA) rats. (**A**) Mechanical nociceptive thresholds were quantified after i.pl. injection of 3 µg CCL2 with prior leukocyte depletion by systemic injection of CTX (filled triangles), 3 µg CCL2 with prior systemic injection of solvent (cyclo-phosphamid (CTX) treatment (open circles), or solvent of CCL2 without CTX treatment (filled circles). CTX injections were performed 3 d with 10 mg/kg and 1d 50 mg/kg before cell and/or CCL2 treatment. (**B**) Representative dot blots (x-axis: forward scatter (FSC) – cell size; y-axis: sideward scatter (SSC) – cell granularity) for leukocyte subpopulations in peripheral blood of untreated (left) and CTX-treated Wistar rats (right) are shown by flow cytometry. Gates were set on beads (for quantification), neutrophils, lymphocytes and monocytes. (**C–F**) Wistar and DA rats were leukocyte-depleted by i.p. injection with CTX followed by i.pl. injection of different numbers of macrophages from either Wistar or DA rats, 30 min later, by i.pl. injection of 3 µg CCL2. Injection of 2×10^6^ macrophages without concomitant injection of CCL2 served as a negative control. (**C**) Wistar rats reconstituted with cells from Wistar rats. I.p. injections of solvent only served as a negative control for CTX treatment (open triangles) with the same injection pattern. (**D**) Wistar rats reconstituted with cells from DA rats. (**E**) DA rats reconstituted with cells from Wistar rats. (**F**) DA rats reconstituted with cells from DA rats. (*p<0.05 compared to time point 0 h, Two Way RM ANOVA, Student-Newman-Keuls, n = 6). Data are presented as means ± SEM.

To circumvent the migratory defect of macrophages from DA rats we examined CCL2-induced hyperalgesia in leukocyte-depleted and reconstituted Wistar and DA rats using local injection of peritoneal macrophages from donor rats. CCL2-induced hyperalgesia in Wistar rats was completely abrogated after depletion of leukocyte cells by pretreatment with cyclophosphamide (CTX) (**Fig. 4A**). CTX treatment resulted in a significant depletion of white blood cells upon CTX treatment on day −3 (100 mg/kg) and day −1 (50 mg/kg) before CCL2 treatment **(** **Fig. 4B** **)**. Total leukocytes were reduced by around 80% (176.0 +/− 23.9 vs. 41.3 +/− 4.9×10^4^ cells/ml). Monocytes were depleted by ca. 70 % (48.8 +/− 10.7 vs. 15.4 +/− 1.0×10^4^ cells/ml), neutrophils by ca. 75 % (45.2 +/− 0.3 vs. 16.6 +/− 2.4×10^4^ cells/ml) and lymphocytes by almost 90 % (81.9 +/− 17.9 vs. 9.1 +/− 1.5×10^4^ cells/ml). CCL2-induced hyperalgesia was reconstituted if macrophages from Wistar rats were injected into the hind paw of leukocyte-depleted Wistar (**Fig. 4C**) or DA rats (**Fig. 4E**). In contrast, adoptive transfer of macrophages from DA rats into leukocyte-depleted Wistar (**Fig. 4D**) or DA rats (**Fig. 4F**) did not induce changes in nociceptive thresholds after CCL2 injection. No effect was observed after CTX pretreatment and i.pl. injection of cells or CTX pretreatment and i.pl. solvent (**Fig. 4C**).

**Figure 5:**
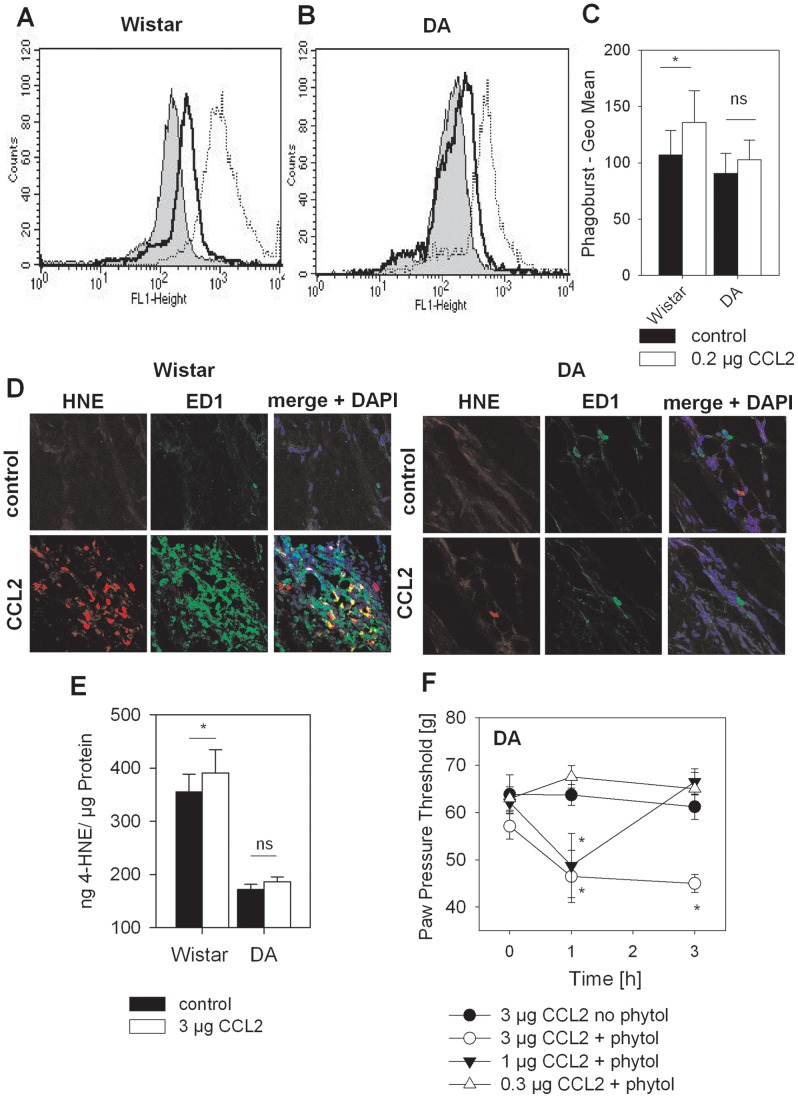
Establishment of CCL2-induced hyperalgesia following restoration of defective ROS generation in Dark Agouti (DA) rats. Peritoneal macrophages from Wistar and DA rats were isolated and treated for 1 h with 0.2 µg CCL2 or solvent control. ROS production was quantified by flow cytometry using the phagoburst assay. Representative histograms are shown for Wistar rats (**A**) and DA rats (**B**) with solvent (grey), CCL2 (black line) or positive assay control PMA (dotted line). (**C**) Results were analyzed statistically (n = 6/group, CCL2 i.pl. (white bars) or solvent (black bars), *p<0.05, t-test). (**D**) Wistar and DA rats were i.pl. injected with CCL2 i.pl. (lower panel) or solvent (upper panels) for 3 h, subcutaneous paw tissue was representative immunohistochemistry is shown for HNE (red), ED1^+^ macrophages (green) and merge of both together with nuclei (DAPI-blue). (**E**) Wistar and DA rats were i.pl. injected with CCL2 i.pl. (white bars) or solvent (black bars) for 3 h, subcutaneous paw tissue was prepared and HNE adducts were quantified by ELISA (n = 6/group, *p<0.05, t-test). (**F**) DA rats were s.c. injected with phytol for 5 days. Mechanical nociceptive thresholds were quantified before and after i.pl. injection of CCL2 (0.3 µg – open circle, 1 µg – filled triangle, 3 µg – open triangle). Control animals were treated 5 days with 0.9 % NaCl before i.pl. injection of 3 µg CCL2 (filled circle), (*p<0.05 versus time point 0 h, Two way RM ANOVA, Student-Newman-Keuls, n = 6). Data are presented as means ± SEM.

To analyze whether CCL2 actually triggered ROS production, oxidative burst was quantified by flow cytometry using DHR 123 oxidation. Macrophages from Wistar rats (**Fig. 5A**), but not DA rats (**Fig. 5B** **)** significantly liberated ROS in response to CCL2 (**Fig. 5C**). HNE adducts are lipid peroxidation products and formed downstream of ROS. Quantification of HNE using ELISA and immunohistochemistry in Wistar rats demonstrated a significantly higher HNE content in CCL2-injected paws (**Fig. 5D, E**). HNE production was mainly seen in ED1^+^ macrophages. However not all ED1^+^ macrophages produced HNE. No increase of HNE content was seen in DA rats treated with CCL2. DA rats in general formed less HNE compared to Wistar rats under baseline conditions. ROS production in DA rats can be reconstituted by pretreatment with phytol [26]. Subcutaneous injection of phytol for 5 days prior resulted in a dose-dependent increase in CCL2-induced hyperalgesia in DA rats (**Fig. 5F**).

### Inhibition of ROS production and scavenging its byproducts in Wistar rats abolishes CCL2-induced hyperalgesia

**Figure 6:**
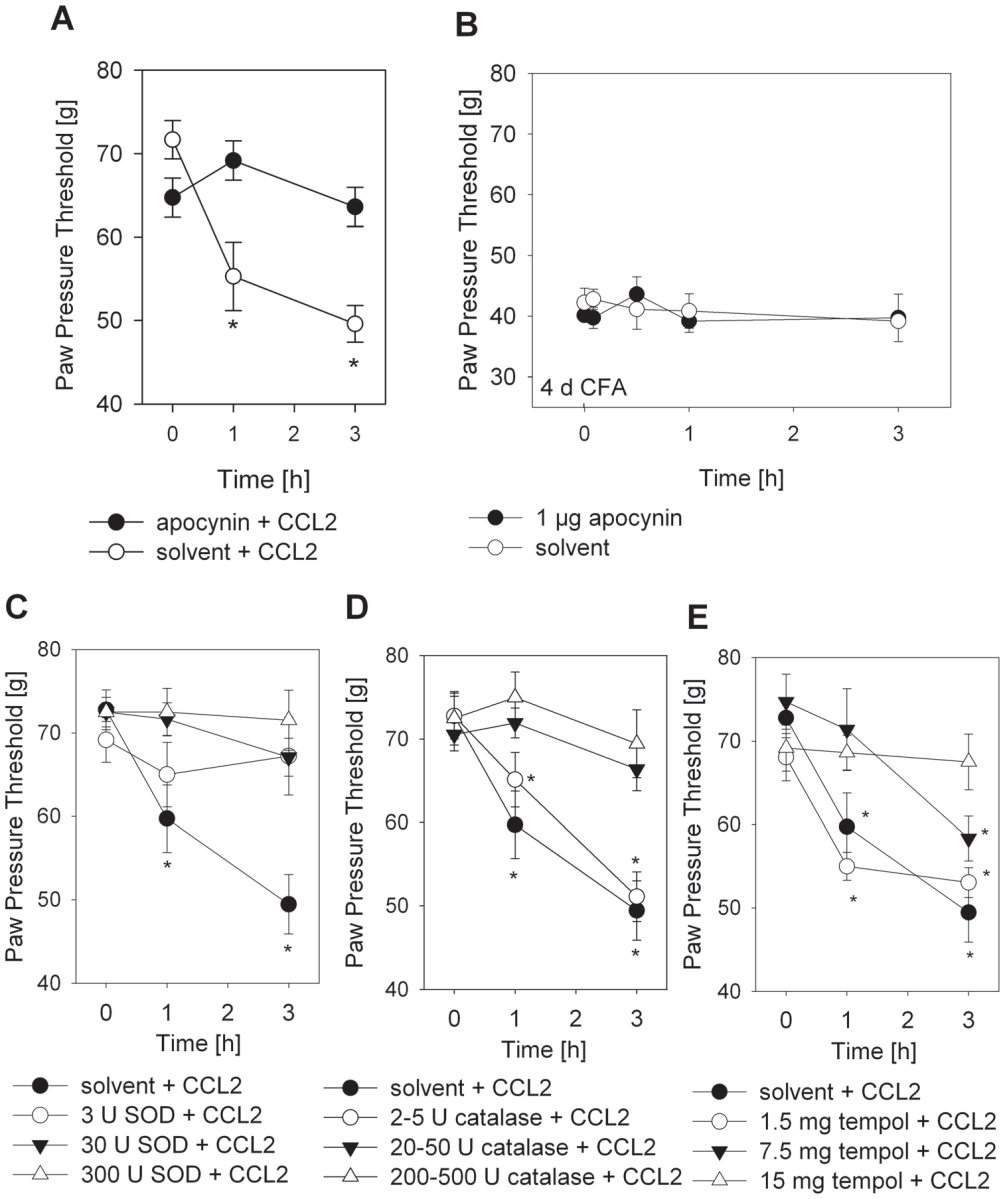
Inhibition of CCL2-induced mechanical hyperalgesia by ROS scavenging in Wistar rats. Mechanical nociceptive thresholds were quantified before and after (**A**) i.pl. injection of 1 µg apocynin with 3 µg CCL2 (filled circles) or 3 µg CCL2 alone and (**B**) i.pl. injection of 1 µg apocynin (filled circles) or its solvent 0.9 % NaCl (open circles) in rats with 4 d CFA inflammation. (**C–E**) Mechanical nociceptive thresholds were quantified before and after i.pl. injection of 3 µg CCL2 alone with the respective solvent (filled circle) or together with (**C**) SOD (3 U open circle, 30 U filled triangle, 300 U open triangle), (**D**) catalase (2–5 U – open circle, 20–50 U – filled triangle, 200–500 U – open triangle), or (**E**) tempol i.p. (1.5 mg – open circle, 7.5 mg – filled triangle, 15 mg – open triangle) (*p<0.05 versus time point 0 h, Two Way RM ANOVA, Student-Newman-Keuls, n = 6). Data are presented as means ± SEM.

In the last step, we further evaluated the contribution of ROS formation to CCL2-induced hyperalgesia. Coinjection of the NADPH oxidase inhibitor, apocynin, blocked CCL2-induced hyperalgesia (**Fig. 6A**). In contrast, treatment with apocynin in 4 d CFA hind paw inflammation did not alter the hyperalgesia in these animals (**Fig. 6B**). We next examined whether inhibition of ROS scavenging enzymes blocks CCL2-induced hyperalgesia in Wistar rats. SOD is an enzyme that catalyzes the conversion of superoxide into oxygen and hydrogen peroxide. CCL2-induced hyperalgesia was completely blocked if SOD was applied together with CCL2 (**Fig. 6C**). Similar effects were observed with catalase, an enzyme that catalyses the decomposition of hydrogen peroxide to water and oxygen (**Fig. 6D**), and with the SOD mimetic tempol, which acts as a membrane permeable superoxide scavenger (**Fig. 6E**).

## Discussion

In the present study, the chemokine CCL2 evoked leukocyte recruitment and hyperalgesia via ROS generation in Wistar rats. In contrast, DA rats with defective NAPDH oxidase activity did not develop CCL2-induced hyperalgesia, but developed hyperalgesia in response to CFA and capsaicin. Adoptive transfer of peritoneal macrophages from Wistar but not from DA rats reconstituted CCL2-induced hyperalgesia in leukocyte-depleted rats from both strains. CCR2 expression as well as *in vitro* migration and CCL2-triggered ROS formation was reduced in peritoneal macrophages in DA rats compared to Wistar. Pharmacological stimulation of ROS production (phytol) made CCL2-induced hyperalgesia possible in DA rats *in vivo*. CCL2-triggered hyperalgesia was fully blocked in Wistar rats by inhibitors of NADPH oxidase or ROS scavenging enzymes. In summary, chemokine (CCL2)-mediated leukocyte recruitment is linked to the induction of hyperalgesia via ROS generation in recruited macrophages.

The role and function of chemokines in the generation of pain is controversial [31]: the neutrophil-recruiting chemokine CXCL1 or CXCL2/3 evokes hyperalgesia [32] in some but not our studies despite selective neutrophil recruitment [1], [33]. In extension of our previous studies, the monocyte-recruiting chemokine CCL2 (despite less leukocyte recruitment) elicited significant hyperalgesia in Wistar rats. We found primarily inflammatory ED1^+^ (CD68^+^) macrophages and some ED2^+^ resident macrophages (data not shown) as seen in the skin [34]. In line with our findings, CCL2 is an important gene up-regulated in various inflammatory and non-inflammatory pain models in rats [35], [36]. In neuropathic pain, the pathogenic role of CCL2 is undisputed due to increased CCR2 expression in nociceptive neurons and CCR2-triggered hyperalgesia by neuronal excitation [6]. In contrast, the molecular link between the chemokine CCL2 and hyperalgesia in the absence of neuropathy is more controversial. We hypothesized that ROS generation could be this molecular link.

To further address the role of ROS generation in CCL2-induced hyperalgesia, we performed experiments in DA rats. DA rats are widely used in arthritis research because of their enhanced susceptibility to arthritis [26] compared to Wistar rats [19] due to defect in oxidative burst in macrophages in response to CCL2 or other stimuli, which was demonstrated here and by others [18]. In our study, DA rats generated comparable mechanical but less thermal hyperalgesia to CFA-induced inflammation compared to Wistar rats. No significant difference in the response to capsaicin treatment in behavior tests or in whole cell recordings of cultured DRG neurons was seen. However, because of the reduced thermal hyperalgesia after CFA in DA rats, a subtle difference in TRPV1 responsiveness is possible which should be examined in further studies. In a sciatic nerve ischemic injury model, DA rats develop less pain-like responses and have a reduced area of intraneural damage as well as higher nociceptive thresholds to heat and mechanical stimulation [37]. In the sciatic nerve ischemic injury model, ROS are supposed to play a role in the pathogenesis of the nerve injury. Taken together, DA rats do not have a general defect in nociceptive function but they are less responsive in selected pain models which involve ROS or thermal inflammatory hyperalgesia.

To explore the reasons for the lack of CCL2-induced hyperalgesia in DA rats, we found a reduced expression of CCR2, reduced cell migration and a reduced ROS formation in response to CCL2. Therefore, we propose that lack of CCL2-induced hyperalgesia was due to reduced CCR2 expression and consecutive defective migratory response and to a deficit in ROS production. *In vitro* chemotaxis experiment effective agonist concentration were observed “nM-pM” range while *in vivo* behaviour experiment effective agonist concentration were seen in “µM” range as observed before [2], [23], [28] possibly due to dilution/degradation in the tissue. Interestingly, several other groups demonstrated that in addition ROS generation is important for chemokine induced chemotaxis and transendothelial migration [38], [39], [40]. Oxidative burst in DA rats can be restored by phytol, a pharmacologic inducer of oxidative burst[26]. Phytol as a side chain of vitamin E is described as an NADPH-oxidase-activating substance that ameliorates the arthritis in DA rats by stimulating ROS production [26], [41]. To sum up, several factors contribute to the lack of CCL2-induced hyperalgesia in DA rats.

Previous studies have indicated that ROS play a major role in the generation of pain. ROS have been implicated in neuropathic pain and significantly contribute to central sensitization [12], [42], [43]. Peripheral ROS generation, e.g. hydrogen peroxide, or downstream products like HNE have also been shown to be involved in the pathophysiology of pain via TRPA1 [44] and TRPV1 [16]. Treatment with antioxidants reduces inflammation-induced mechanical and thermal pain thresholds [11]. In our study intraplantar CCL2 injection resulted in increased levels of HNE in macrophages in Wistar rats. More importantly, blockage of ROS formation using an NAPDH oxidase inhibitor apocynin, or enhancement of ROS degradation using SOD, catalase or tempol completely inhibited the CCL2-triggered hyperalgesia in Wistar rats. On the contrary, in later stages of CFA inflammation the NAPDH oxidase inhibitor apocynin could not reverse the hyperalgesia in line with reduced CFA-elicited thermal hyperalgesia in DA rats. Summarized, CCL2-induced hyperalgesia is critically dependent on ROS. This is in line with other studies pointing to a pathogenic role of ROS in several models of pain.

In conclusion, CCL2 generates less thermal and mechanical pain in DA compared to Wistar rats due to a lack p47^phox^, a member of the NADPH oxidase complex, and a defect in CCR2 expression in macrophages. The monocyte/macrophage attracting chemokine CCL2 directly triggered ROS via NADPH oxidase. This was causally linked to the development of hyperalgesia. In summary, we described ROS as the molecular link between monocyte chemotaxis and inflammatory pain. A better understanding of the molecular mechanisms of leukocyte recruitment, activation and sensitization of nociceptors could potentially provide new therapeutic approaches for the treatment of inflammatory pain. This could be useful for both patients with postoperative pain and for those with inflammatory rheumatic diseases (e.g. rheumatoid arthritis).
